# Systematic Review and Meta-Analysis on the Impact of Thrombolytic Therapy in Liver Transplantation Following Donation after Circulatory Death

**DOI:** 10.3390/jcm7110425

**Published:** 2018-11-08

**Authors:** Kumar Jayant, Isabella Reccia, Francesco Virdis, A. M. James Shapiro

**Affiliations:** 1 Department of Surgery and Cancer, Imperial College London, London W12 OHS, UK; Isabella.Reccia@imperial.nhs.uk; 2 Department of Surgery, Kings College, London SE5 9RS, UK; francesco.virdis@hotmail.it; 3 Department of Surgery, University of Alberta, Edmonton, AB T6G 2B7, Canada; amjs68@gmail.com

**Keywords:** liver transplant, thrombolytic, tissue plasminogen activator, donor after cardiac death

## Abstract

Aim: The livers from DCD (donation after cardiac death) donations are often envisaged as a possible option to bridge the gap between the availability and increasing demand of organs for liver transplantation. However, DCD livers possess a heightened risk for complications and represent a formidable management challenge. The aim of this study was to evaluate the effects of thrombolytic flush in DCD liver transplantation. Methods: An extensive search of the literature database was made on MEDLINE, EMBASE, Cochrane, Crossref, Scopus databases, and clinical trial registry on 20 September 2018 to assess the role of thrombolytic tissue plasminogen activator (tPA) flush in DCD liver transplantation. Results: A total of four studies with 249 patients in the tPA group and 178 patients in the non-tPA group were included. The pooled data revealed a significant decrease in ischemic-type biliary lesions (ITBLs) (*P* = 0.04), re-transplantation rate (*P* = 0.0001), and no increased requirement of blood transfusion (*P* = 0.16) with a better one year graft survival (*P* = 0.02). Conclusions: To recapitulate, tPA in DCD liver transplantation decreased the incidence of ITBLs, re-transplantation and markedly improved 1-year graft survival, without any increased risk for blood transfusion, hence it has potential to expand the boundaries of DCD liver transplantation.

## 1. Introduction

Liver transplantation has been established as a life-saving therapy and mainstay treatment for all forms of end-stage liver disease. However, its success is thwarted by the limited availability of donor organs and the increase in the incidence of liver diseases [1,2]. A recent report from the 2017 United States organ procurement and transplantation network has revealed some dispiriting data. Almost 3000 patients dropped-off the liver transplant waiting list due to death or extreme sickness [2]. However, in the present circumstances, it seems to be an inevitable corollary secondary to worsening disease during the waitlist period. To counter the paucity of organs, transplant centers have broadened the graft acceptance criteria to incorporate high risk, extended criteria donors (ECDs), including organs from donor after cardiac death (DCD) donors, despite the fact that such livers have an increased risk for complications. Halpern et al. outlined that the apt and timely call for organ procurement from DCD can underpin the organ pool by providing a 10% and 25% increase in suitable and marginal transplant candidates, respectively [3]. In recent years, the use of DCD liver donors has risen steadily and accounts for 5–10% of all liver transplants performed in North America [4]. In spite of these promising trends, the overzealous use of DCD donors has been trounced by the high risks of dispiriting outcomes like biliary complications, hepatic artery thrombosis, and primary nonfunction (PNF) [5,6,7]. Analysis of the data from the Scientific Registry of Transplant Recipients (SRTR) reported inferior post-transplantation outcomes with the DCD group over donation after brain death (DBD) due to poor graft quality [6].

**Figure 1:**
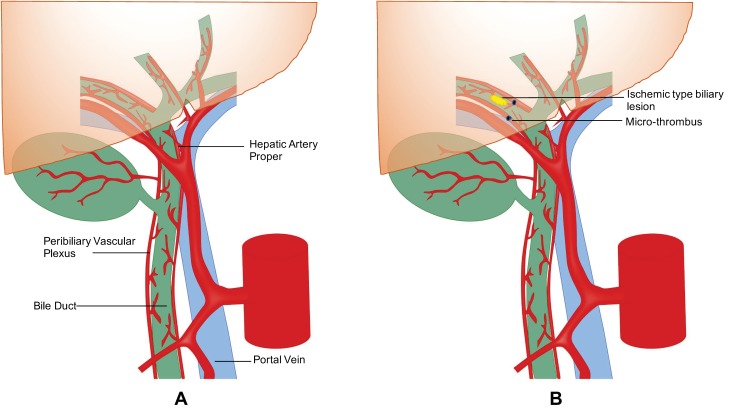
(**A**): Pictorial representation peribiliary vascular plexus. (**B**): Pictorial representation of the development of micro-thrombus in peribiliary plexus.

Biliary complications such as ischemic-type biliary lesions (ITBLs), or ischemic cholangiopathy (IC), are seen in up to 30–50% of cases and are considered the major determinant of poor outcomes in DCD liver transplantation [7,8,9]. These biliary lesions may be focal or diffuse, progressive, and respond poorly to endoscopic/radiologic interventions, consequently leading to cholestatic liver failure requiring re-transplantation. The critical risk factors leading to the development of ITBLs following DCD liver transplantation include prolonged cold ischemia time (CIT), donor warm ischemia time (WIT), and elderly donors [10,11]. The exact reason for the higher susceptibility of ITBLs in DCD liver is unclear, but is likely underpinned by disruption and microthrombi formation within the biliary ductal microcirculation. Hepatic parenchyma has a dual blood supply from the hepatic artery and portal vein. However, the blood supply to the intrahepatic and extrahepatic biliary system of transplanted liver is exclusively derived from the hepatic artery via the peribiliary plexus [12,13]. The blood vessels plexus first supplies the outer layer of the bile duct, hence the peribiliary glands situated in the periphery are less prone to injury than the endoluminal biliary epithelium [14]. Almost 92% of epithelium on the luminal aspect of the bile duct, compared to 17.5% of the peribiliary glands, becomes damaged after cold preservation of the donor liver [15]. The peribiliary glands are the niche of biliary progenitor cells and aid the regeneration of biliary epithelium; however, in cases of severe damage to these glands, the incidence of non-anastomotic stricture (NAS) is high [15]. The obligatory acirculatory phase encountered during donor WIT promotes stasis of blood and microthrombi formation in the peribiliary microcirculation which can disrupt the delicate blood supply to the bile duct leading to ischemia, fibrosis, and stricture formation [16,17], see Figure 1.

Hashimoto et al. (2010) postulated that administration of a thrombolytic agent (thrombolytic flush), tissue plasminogen activator (tPA), into the hepatic artery prior to reperfusion may dissolve microthrombi in the biliary microcirculation and thereby mitigate risk for the development of ITBLs [18,19,20]. Early results prompted a shift in clinical practice with a minority of North American centers adopting this practice in DCD donation. Various protocols differ with respect to dose, timing, and the type of thrombolytic flush given either during procurement (tPA intervention at procurement) or into the hepatic artery while implanting the donor liver before restoring portal vein perfusion (tPA intervention at implantation). Proponents of administering tPA to the donor argue that more effective thrombolysis may be achieved and that by giving tPA to the donor and not to the recipient, carry-over and risk of severe bleeding in the recipient is mitigated [21]. In contrast, advocates of administration in the recipient argue that fibrinolytic activity occurs upon portal vein reperfusion with the influx of plasminogen and return of normothermia. Thus, the administration of tPA while implanting ensures its optimal functioning as hypothermia severely dampens its fibrinolytic efficacy [22,23,24]. It is important to recognize that tPA and other thrombolytics are not metabolically active or effective in the cold phase. This is clearly evident from the study of Pietersen et al., 2016, [25] where liver was flushed with thrombolytic agent urokinase, although the study did not demonstrate any benefit as a nonanastomotic biliary structure. However, this could be explained by virtue of the use of urokinase at a cold temperature during backbench preparation as the fibrinolytic activity of urokinase declines with a lowering of temperature [26].

Potentially, tPA could be administered during normothermic ex vivo perfusion if that technology is adopted. However, the increased risk of bleeding on the device in the absence of any circulating clotting factors remains untested.

Despite a hesitant increase in the adoption of thrombolytic protocols by transplant centers, the available literature is limited to determine its safety and efficacy in DCD liver transplantation. The present study aims to systematically review the literature and where possible statistically compare the available data to compare outcomes for rates of ITBLs, biliary complications, hepatic artery thrombosis (HAT), graft and patient survival, and blood transfusion following administration of thrombolytic flush in DCD liver transplantation.

## 2. Methods

### 2.1. Search Strategy

The present meta-analysis was performed after completion of registration (CRD42018102958) in PROSPERO, an international database of prospectively registered systematic reviews. The search strategy designed according to the guidelines mentioned in the Cochrane Handbook for Systematic Reviews of Interventions and reported as per the guidelines proposed by a meta-analysis of observational studies in epidemiology [27,28]. 

A detailed literature search was carried out, on the role of thrombolytic (tPA) flush in DCD liver transplantation on MEDLINE, EMBASE, Cochrane, Crossref, Scopus, and clinical trial registries on 20 September, 2018.

The search covered a period from 2010 (the year of the first reported use of tPA in DCD liver transplant) to September 2018 [20]. The medical subject headings (MeSH) terms and free text words were searched in various permutations and combinations: “liver transplantation”, AND “donation after cardiac death” OR “DCD” OR “donation after circulatory death” OR “non-heart beating death” AND “thrombolytic therapy” OR “tissue plasminogen activator” OR “tPA” were searched with adaption to each database without any limitation to complete the analysis. Further, a manual search for conference abstracts, bibliographies, and a citation list of the relevant articles were examined for additional study.

### 2.2. Inclusion Criteria

Both retrospective and prospective studies available in the literature comparing thrombolytic (tPA) flush with no tPA in DCD liver transplant were included. All other studies or publications such as editorials, reviews, and letters were excluded. The outcomes of interest were adverse events, ITBLs, biliary complications, hepatic artery thrombosis, blood transfusion, and patient and graft survival, as shown in Table 1.

### 2.3. Data Extraction

**Figure 2:**
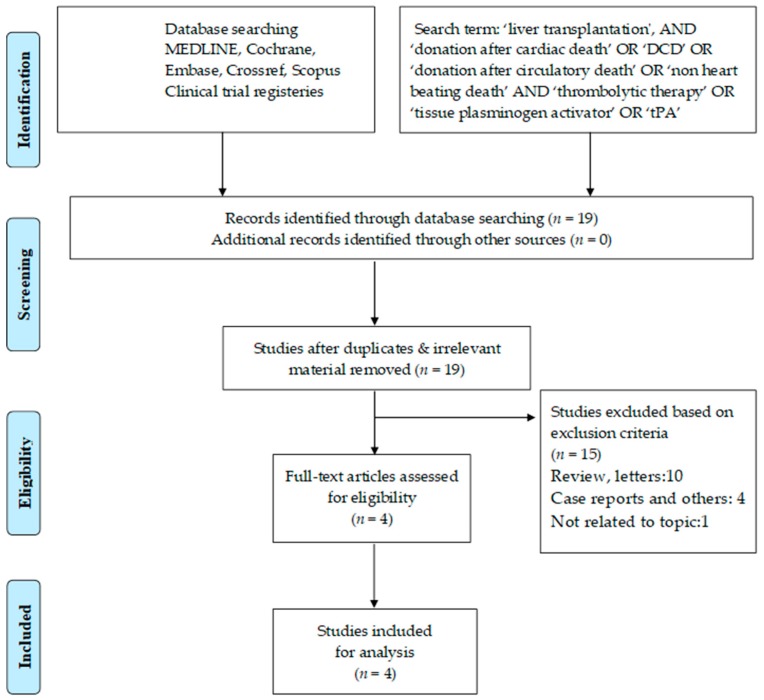
Search strategy and study selection used in this systematic review as per the Preferred Reporting Items for Systematic Reviews and Meta-analyses (PRISMA) protocol.

Two separate physician reviewers, KJ and AMJS, employed a two-stage method to conduct study screening independently. At the first stage, titles and abstracts were scrutinized for the purpose of excluding obviously ineligible studies. At the second stage, the full texts or limited text (posters) were read and further exclusion of any ineligible studies was made. In case of disagreements, matters were resolved via discussion until a consensus was achieved. The Preferred Reporting Items for Systematic Reviews and Meta-analyses (PRISMA) guidelines were used to complete the search strategy and study selection, see Figure 2.

### 2.4. Statistical Analysis

The validity of pre-specified inclusion and exclusion criteria of the included studies was determined by using the Cochrane Risk of Bias tool. Each study was thoroughly analyzed to evaluate the above-mentioned parameters, see Table 2. The Cochrane Collaboration Review Manager (RevMan) version 5.3 can analyze a minimum of two trials with the available continuous and dichotomous data. The effect measures used were mean difference (MD) for continuous data and odds ratio (OR) for dichotomous data, with 95% confidence intervals (CI). In the case of continuous data presented as median and range, the statistical methods described by Hozo et al. were applied to calculate the mean and standard deviation [29].

The heterogeneity (*I*^2^) between the trials was considered low with an *I*^2^ value ≤25%, moderate with an *I*^2^ value >25% but <75%, and high with an *I*^2^ value of ≥75%. An *I*^2^ statistic of more than 30% was determined to be significant. In the case of significant heterogeneity, the random effects model assessment was used following the evaluation of the forest plot while a fixed-effect model was applied in the situation of low heterogeneity [30,31]. Unfortunately, publication bias could not be assessed in the present study as it requires at least 10 trials to assess it and our current meta-analysis involved only four trials [32].

### 2.5. Protocols for the Use of Thrombolytic (tPA) in DCD Liver Transplant

**Figure 3:**
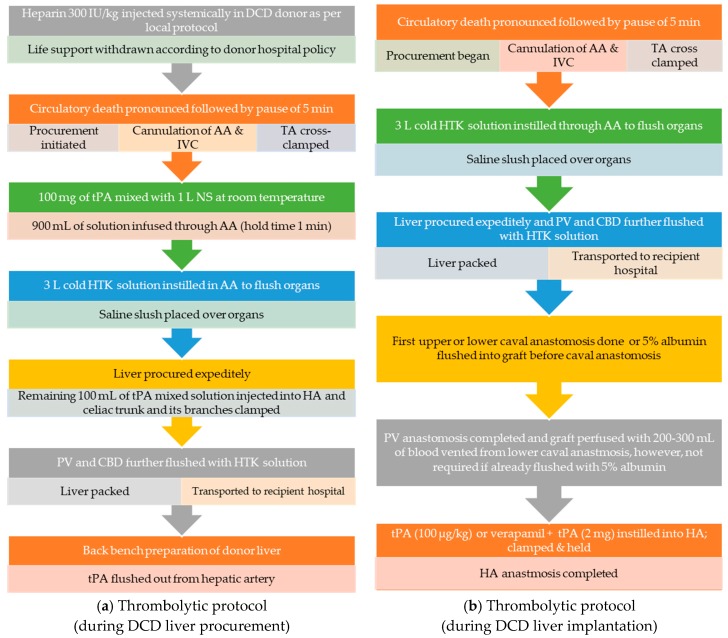
(**a**): Thrombolytic (tPA) protocol during DCD liver procurement. (**b**): Thrombolytic (tPA) protocol during implantation of DCD liver. Abbreviations: AA, abdominal aorta; CBD, common bile duct; HA, hepatic artery, HTK, Histidine-tryptophan-ketoglutarate; IVC, inferior vena cava; NS, normal saline; PV, portal vein; TA, thoracic aorta; tPA, tissue plasminogen activator.

The protocols available in the literature describing dosing and administration of the thrombolytic agent, tPA, in DCD liver transplantation are outlined below in Figure 3a,b [18,23,24,33]. Thrombolytics are known to dissolve thrombi and recanalize occluded blood vessels; native tissue plasminogen activator (tPA) has a half-life of only 5 min, a narrow therapeutic index, and a temperature-dependent efficacy [34]. Meunier et al. (2012) demonstrated the maximum mean fractional clot loss at 37 °C; however, decreasing the temperature to 30 °C retained up to 75% of activity and can deliver optimal clot lysis [22].

#### 2.5.1. Thrombolytic (tPA) Protocol during DCD Liver Procurement

Where thrombolytic therapies are administered at the earliest time-point during the donor flush, 100 mg of tPA is instilled into 1 L of normal saline (NS) at room temperature. The cannulation of abdominal aorta and cross-clamp of thoracic aorta are followed by the administration of 900 mL of tPA mixed solution, which is held for 1 min to ensure uniform distribution of the drug in the hepatic vasculature. It is important to know that thrombolytic flush is given before administrating the cold preservation solution which provides the optimum temperature and ensures maximum efficacy. Subsequently, 3–4 L of cold preservation solution, histidine-tryptophan-ketoglutarate (HTK) is flushed into the abdominal aorta and the procurement of liver is completed. The remaining 100 mL of tPA mixed solution is injected into the hepatic artery and microvascular clamps are applied on the celiac trunk and its branches. An additional 500 and 100 mL of cold HTK solution is flushed in the portal vein and bile duct, respectively. The organ is packed and transported to the recipient’s hospital, where tPA is flushed out from the hepatic artery during back bench preparation.

#### 2.5.2. Details of Thrombolytic (tPA) Protocol during DCD Liver Implantation

The efficacy of tPA is determined by its ability to convert circulatory plasminogen to plasmin that lyse the thrombus. The donor liver has low plasminogen levels and is preserved in the hypothermic state which further impedes tPA activity [35,36]. Currently, most centers adopting thrombolytic therapy in DCD liver transplantation favor administration after the restoration of portal flow, which not only leads to the influx of plasminogen but also helps in achieving therapeutic efficacy by raising the temperature.

There are two approaches regarding the dosage and timing of the administration of thrombolytic during the implantation of a donor’s liver. The first calculates the tPA dose based on the donor’s weight (100 µg/kg). Thrombolytic is injected into the hepatic artery above the origin of the gastroduodenal artery after the completion of upper and lower caval anastomosis and the placement of corner sutures for portal vein anastomosis. Vascular bulldog clips are applied to promote an even dispersion of drug into the intrahepatic vasculature. The liver may be perfused with 200–300 mL of blood vented from lower caval anastomosis before the restoration of portal circulation. The hepatic artery is anastomosed to complete the vascular reperfusion. 

The alternative approach, promoted by a group in Toronto [23], eliminates the need for portal blood flush before restoring perfusion and involves injecting a fixed dose of tPA (2 mg) into the hepatic artery. Prior to the completion of upper and lower caval anastomosis, the graft is perfused with 5% albumin and the portal vein is anastomosed. Subsequently, a vasodilator (verapamil, 5 mg) is injected into the hepatic artery followed by a fixed dose of tPA (2 mg) and the vascular clamp is placed as described earlier. After holding the thrombolytic for few minutes, arterial anastomosis is completed.

## 3. Results

### 3.1. Search Results

The primary literature search yielded a total of 19 manuscripts; of these, 15 articles were excluded following careful evaluation of previously described selection criteria. After the resolution of differences between reviewers, a total of four studies were retrieved for further review and data extraction [21,23,24,33]. These include, three published papers on retrospective studies and one conference abstract with a prospective randomized/non-randomized study, see Table 2. The detailed data of all the studies related to the adverse events, ITBLs, biliary complications, hepatic artery thrombosis, the requirement of blood transfusion, and patient and graft survival are summarized in Table 2 and Table 3. The detail results of these data analyses are structured below.

### 3.2. Biliary Complications.

**Figure 4:**
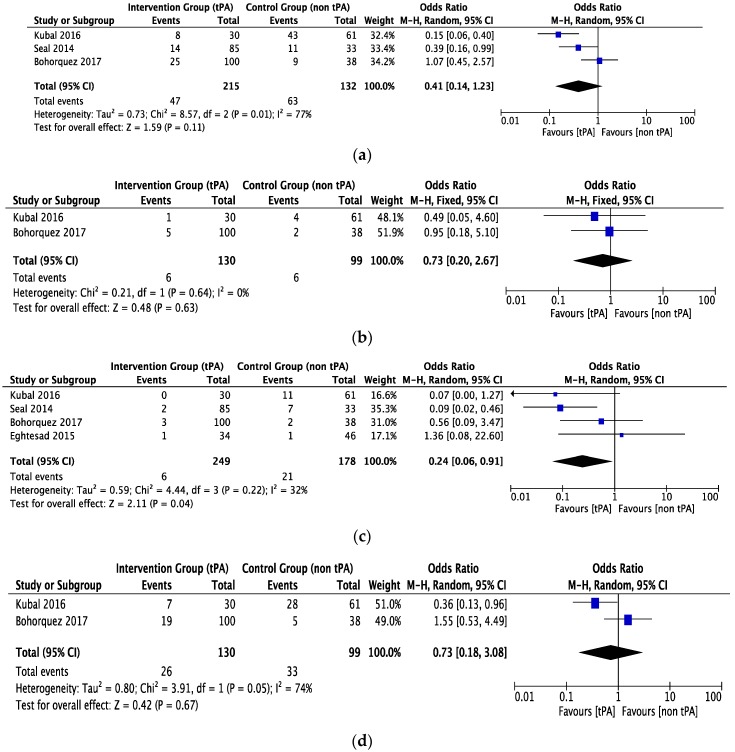
The forest plot represents, (**a**) the total biliary complication in DCD liver recipients with or without prior thrombolytic (tPA) flush. The size of the squares depicts the effects while comparing the weight of the study in the meta-analysis. The diamond doesn’t suggest any significant difference following random effects analysis. A 95% confidence interval is represented by horizontal bars. (**b**) The bile leak in DCD liver recipients with or without prior thrombolytic (tPA) flush. The size of the squares depicts the effects while comparing the weight of the study in the meta-analysis. The diamond doesn’t suggest any significant difference following fixed effects analysis. A 95% confidence interval is represented by horizontal bars. (**c**) The ischemic-type biliary lesions (ITBLs) in DCD liver recipients with or without prior thrombolytic (tPA) flush. The size of the squares depicts the effects while comparing the weight of the study in the meta-analysis. The diamond shows the significant favor towards the tPA flush group following random effects analysis. A 95% confidence interval is represented by horizontal bars. (**d**) The anastomotic biliary stricture in DCD liver recipients with or without prior thrombolytic (tPA) flush. The size of squares depicts the effects while comparing the weight of the study in the meta-analysis. The summary effect (diamond) doesn’t suggest any significant difference following fixed effects analysis. A 95% confidence interval is represented by horizontal bars; DCD, donor after circulatory death; tPA, tissue plasminogen activator; ITBLs, ischemic-type biliary lesions.

Total biliary complications were reported by three studies [21,23,24] with 215 patients in the tPA flush or thrombolytic group and 132 patients in the non-tPA group. The observed heterogeneity was high with no significant difference in total biliary complications in either of the groups (OR = 0.41, CI 0.14 to 1.23, *P* = 0.11, *I*^2^ = 77%), see Figure 4a. Both groups had similar rates of bile leak (two studies, 229 patients, OR = 0.73, CI 0.20 to 2.67, *P* = 0.63, *I*^2^ = 0%), see Figure 4b.

Rates of ischemic-type biliary lesions (ITBLs) were reported in four studies [21,23,24,33] with 249 patients in the tPA group, 178 patients in the non-tPA group, and a heterogeneity of 32%. The pooled data showed a significant reduction in ITBLs in the tPA group compared to the non-tPA group (OR = 0.24, CI 0.06 to 0.91, *P* = 0.04, *I*^2^ = 32%), see Figure 4c. Anastomotic biliary strictures were reported by two studies [23,26] with a moderate heterogeneity between studies. However, there was no difference between the two groups (OR = 0.73, CI 0.18 to 3.08, *P* = 0.67, *I*^2^ = 74%), as shown in Figure 4d.

### 3.3. Other Complications

**Figure 5:**
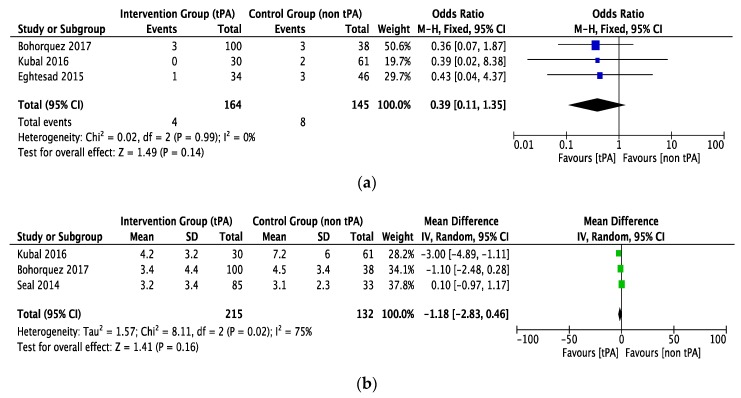

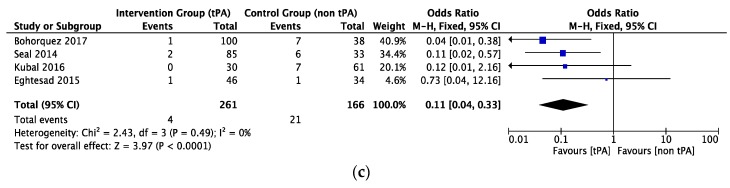
The forest plot represents, (**a**) the HAT in DCD liver recipients with or without prior thrombolytic (tPA) flush. The size of the squares depicts the effects while comparing the weight of the study in the meta-analysis. The diamond doesn’t suggest any significant difference following random effects analysis. A 95% confidence interval is represented by horizontal bars. (**b**) The blood transfusion (pRBC units) in DCD liver recipients with or without prior thrombolytic (tPA) flush. The size of squares depicts the effects while comparing the weight of the study in the meta-analysis. The diamond doesn’t suggest any significant difference following random effects analysis. A 95% confidence interval is represented in horizontal bars. (**c**) Re-transplantation in DCD liver recipients with or without prior thrombolytic (tPA) flush. The size of the squares depicts the effects while comparing the weight of the study in the meta-analysis. The diamond shows the significant favors towards the tPA flush group following fixed effects analysis as applicable. A 95% confidence interval is represented in horizontal bars; DCD, donor after circulatory death; HAT, hepatic artery thrombus; tPA, tissue plasminogen activator; pRBC, packed red blood cells.

The rate of hepatic artery thrombosis was comparable in both groups (OR = 0.39, CI 0.11 to 1.35, *P* = 0.14, *I*^2^ = 0%), as shown in Figure 5a. This outcome was determined by three studies [21,24,33] with a low heterogeneity between them. The tPA and non-tPA groups included 164 and 145 patients, respectively.

Three studies reported blood transfusion (units of pRBC received) in the two groups with a high heterogeneity between studies. Both groups received statistically equivalent amounts of transfusion (Mean difference (MD) = −1.18, CI −2.83 to 0.46, *P* = 0.16, *I*^2^ = 75%), see Figure 5b.

Rates of listing or completion of re-transplantation were reported in four studies [21,23,24,33], with 261 patients in the tPA group, 166 patients in the non-tPA group, and a low heterogeneity. Patients from the tPA group had significantly lower re-transplantation rates (OR = 0.11, CI 0.04 to 0.33, *P* < 0.0001, *I*^2^ = 0%), see Figure 5c.

### 3.4. Survival

**Figure 6:**
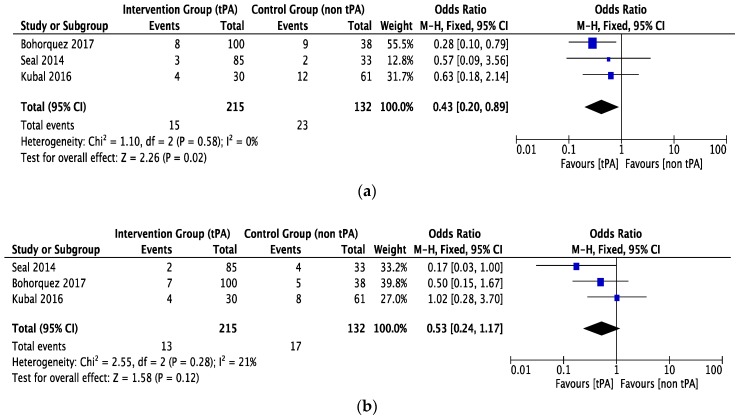
The forest plot represents, (**a**) graft survival in DCD liver recipients with or without prior thrombolytic (tPA) flush. The size of the squares depicts the effects while comparing the weight of the study in the meta-analysis. The diamond does suggest significant favor towards the tPA flush group following random effects analysis as applicable. (**b**) Patient survival in DCD liver recipients with or without prior thrombolytic (tPA) flush. The size of the squares depicts the effects while comparing the weight of the study in the meta-analysis. The diamond doesn’t suggest any significant favor towards the tPA flush group following fixed effects analysis as applicable. A 95% confidence interval is represented by horizontal bars; DCD, donor after circulatory death; tPA, tissue plasminogen activator.

One-year graft survival was determined by three studies [21,23,24] with 215 patients in the tPA group and 132 in the non-tPA group. There was a low statistical heterogeneity between studies. Fixed-effect model analysis demonstrated significantly better graft survival in the tPA group (OR = 0.43, CI 0.20 to 0.89, *P* = 0.02, *I*^2^ = 0%), see Figure 6a. 

Three studies [21,23,24] with low statistical heterogeneity reported one-year patient survival, which was found to be comparable in both groups (OR = 0.53, CI 0.24 to 1.17, *P* = 0.12, *I*^2^ = 21%), see Figure 6b.

## 4. Discussion

In the past decade, there has been an increasing trend towards DCD liver transplantation. However, the data suggest that despite the introduction of a number of innovative approaches, DCD recipients encounter inferior graft survival rates, mainly related to higher rates of PNF, early graft dysfunction (EGD), and biliary complications, particularly ITBLs. Several institutions and regulatory agencies have raised concerns surrounding the 1.5–2 times elevated risk of graft loss and the 3 times elevated risk of re-transplantation, which leads to a persistent reluctance among some clinicians to justify an expanded adoption of DCD livers [37,38,39]. The biliary complications, notably ITBLs, are implicated as the prime reason for worse outcomes leading to a prolonged hospital stay, frequent invasive biliary interventions (2.4–8.1 procedures), and higher readmission rate within a year of implantation [40]. Multiple single-centers’ retrospective studies and a randomized trial assessed the applicability of a thrombolytic agent in DCD liver transplants. However, the association is rather underpowered owing to the small sample size, difference in donor and recipient selection, surgical technique, and post-operative medical management. 

To our knowledge, this is the first meta-analysis assessing the impact of a thrombolytic agent in DCD liver transplantation and outlines the broader picture of this practice with a primary focus on biliary complications and survival outcomes. The data analysis was conducted using a rigorous methodology which led to a sample size of 261 DCD livers subsequent to thrombolytic pre-treatment relative to 166 conventional DCD recipients and demonstrated intriguing results. Further, in the discussion we have highlighted our findings and the impact of tPA flush in liver transplant surgery.

ITBLs and biliary sequelae have been frequently implicated in the increased morbidity and mortality after liver transplantation. The reported incidence of ITBLs in the DCD group was 16% compared to 3% in DBD recipients [40]. A recent meta-analysis by Tang et al. (2018) included fourteen studies and 4610 patients and concluded that the biliary complications were significantly higher (2.5 times) in DCD liver transplant compared to DBD recipients. Further, the ITBLs inferred from the analysis of thirteen studies including 3875 transplant recipients showed a significantly higher incidence in DCD population [41]. ITBLs can lead to chronic, incurable biliary complications with potentially devastating implications on the post-transplant quality of life [42,43]. The pooled data of our meta-analysis clearly demonstrates a significant reduction in ITBLs following the thrombolytic therapy in DCD liver transplantation, and that this may be applied safely without risk of excess recipient bleeding. Moreover, Seal et al. [23] outlined a significantly lower incidence of intrahepatic strictures following thrombolytic application in the DCD group. However, no such difference was noted while comparing extrahepatic stricture.

Hepatic artery thrombosis (HAT) is considered to be a rare but critical reason for graft loss and mortality following liver transplantation and is seen more frequently with DCD than DBD liver transplants [41,44]. The prolonged period of donor warm ischemia has a deleterious effect on vascular endothelium leading to the loss of surface glycosaminoglycan expression and thereby may incite microthrombus formation. Review of the available literature in the present meta-analysis has outlined a significant decrease in HAT with the application of thrombolytic agents as an adjunct to DCD donor preemptive heparin administration where allowed by local practice, in order to lower risk of thrombosis [45].

Several database analyses have demonstrated inferior graft and patient survival following DCD liver transplantations. Selck et al. evaluated graft survival in DCD liver and reported 1-year, 2-year, and 3-year graft survivals of 72%, 63%, and 57%, respectively, in comparison to 84%, 78%, and 74%, respectively, for DBD liver transplants (*P* < 0.001) [46]. Merion et al. assessed the graft failure rate and revealed an 85% higher risk in DCD liver transplant. The authors also reported a 1-year survival rate of 70.1% and a 2-year rate of 60.5% for DCD livers as opposed to 83.0%, and 75.0% for DBD (*P* < 0.001) [39]. Various single-centers have studied patient survival in DCD liver transplants. Foley et al. reported a diminished 1-year, and 2-year patient survival of 80% and 68% with regard to DCD, while the 1-year and 2-year patient survival rate for DBD was 91% and 84%, respectively (*P* = 0.002). The authors further mentioned a reduced graft survival of 67% and 56% with DCD in contrast to 86% and 80% with DBD (*P* = 0.0001) [47]. In contrast, Skaro et al. found no difference in patient survival, but a reduced 1- and 3-years graft survival for DCD recipients of 61.3% and 52.6%, and 85.2% and 74.2% for DBD (*P* = 0.005). In addition, the authors mentioned a 3.2-fold increased risk of re-transplantation among DCD recipients [40]. The recipients of DCD liver have a higher rate of re-transplantation and reduced survival, which suggests that the earliest possible administration of thrombolytics might offer the best protection. However, a major draw-back of administration in DCD donors is that tPA cannot be administered ethically until death is confirmed and the legal stand-off period has been instigated. Therefore, at the time tPA is given and the liver is being cooled, the enzymatic activity of tPA is markedly suppressed to the point of potential futility. The current meta-analysis revealed a significantly better 1-year graft survival and a reduced requirement of re-transplantation in all thrombolytic groups.

The reduced risk of ITBLs and associated complications with improved graft survival without increased risk for blood transfusion following thrombolytic flush of DCD liver could also be influenced by other risk factors including prolonged donor WIT and CIT, bile-induced epithelial damage, and the use of donors >40 years [48,49,50]. A recent meta-analysis by Cao et al. (2016) involving 23 studies and 1184 DCD patients stated that changing the location of life support withdrawal to the operation theatre brought a significant reduction in donor WIT, which translated into reduced biliary complications and improved graft and patient survivals [51]. In addition, another crucial determinant for the development of ITBLs is CIT, with studies showing that 6 to 10 h of CIT may result in increased graft failure risk compared to those with CIT less than 6 h. Hence, attempts to minimize CIT may limit biliary complications and improve the successful utilization of DCD donor livers [52,53]. In the absence of a multivariate analysis, we cannot identify the precise effect size and the influence of these determinants on outcomes associated with thrombolytic pre-treatment in DCD liver transplant, hence, further research is required to elucidate this analogy.

Another important point to be considered is that studies included in this meta-analysis focused on the study population who met the inclusion criteria, making them an excellent source to determine the potential benefit of thrombolytic therapy in DCD livers. The pooled data analysis showed no difference between either group in terms of donor WIT and CIT. The evidence gathered has clearly demonstrated the benefits of thrombolytic therapy in DCD liver transplant, which can potentially decrease the incidence of post-transplant complications and the waiting list.

Given the outcomes of the present meta-analysis, the application of thrombolytic therapy is recommended [18,21,23,24], while, others are contentious about the possible role of tPA due to various reasons and consider it to be a wondrous fable. The hyperfibrinolytic state and low incidence (2.7%) of thrombosis and the minuscule amount of microthrombi found in the peribiliary vascular plexus after cold storage, make thrombolysis a futile and potentially hazardous practice [15,54]. Hashimoto et al. reported excessive post-reperfusion hemorrhage in 64% of patients receiving DCD liver following tPA flush. However, this was not a dose-dependent risk but rather an exaggerated reperfusion effect secondary to poor graft characteristics such as donors > 40 years, high BMI (>30), and prior abdominal surgery [18]. Later the DCD donor’s exclusion criterion for thrombolytic flush was modified and the risk of excess bleeding was mitigated. However, additional measures were implemented to minimize the introduction of a drug into systemic circulation such as tPA flush before implanting the liver or ensuring the removal of the drug through back bleeding [18,21,23,24].

The merits of using thrombolytic therapy in DCD liver transplant have shifted the focus from theoretical risk to observed clinical benefits and paved the way to change the protocol for DCD liver transplantation in a limited number of centers. Financial analysis indicates that DCD recipients incur a greater hospital cost per year of life, with the largest portion attributable to biliary complications, especially non-anastomotic strictures and re-transplantations. An average increase of almost 80,000 USD has been demonstrated in the setting of biliary strictures. Moreover, re-transplantation is associated with increased costs of 180,000 USD, based on 2016 data [55]. The pooled data of the present meta-analysis indicated that thrombolytic therapy in DCD livers has a positive influence on re-transplantation and graft survival. However, the influence of other risk factors such as prolonged donor WIT and CIT, bile-induced epithelial damage, donors >40 years, surgical skills, postoperative care, and hospital discharge protocols cannot be excluded.

The index meta-analysis has a few limitations, which must be acknowledged, especially due to clinical heterogeneity of the included studies. The random effects model for pooled data analysis was used to limit the shadow of heterogeneity. A publication bias could not be excluded because of the limited number of included studies. Here, we could only identify four trials and thus further large-scale trials would provide much-needed data to allow firmer conclusions, regarding the role of thrombolytic flush in DCD liver transplantation. However, considering the costs and ethical concerns, the suitability of conducting such a study is a matter of debate. Despite these limitations, this meta-analysis has outlined the benefits and safety issues associated with the application of thrombolytic (tPA) flush in DCD liver transplantation.

## 5. Conclusions

The application of thrombolytic therapies in DCD liver transplantation decreased ITBLs, HAT, and re-transplantation with markedly improved 1-year graft survival, without an increased risk for blood transfusion. The most intriguing advantage of thrombolytic flush is its potential to expand the boundaries of DCD liver transplantation. Despite the difference in protocols and dosing techniques, the present evidence in the available literature does suggest the benefit of tPA in preventions of ITBLs in DCD liver transplantation. However, continued research is necessary to determine the optimum protocol of thrombolytic flush and establish universal guidelines and indications for its broader implementation.

## Figures and Tables

**Table 1:** Criteria for the inclusion of studies.

Parameters	Details
Study design	Retrospective, prospective, randomized, or non-randomized
Study group	DCD Liver transplant
Study size	Any
Length of follow-up	Any
Source	Peer-reviewed journals, posters
Language	Any
Outcome measure	ITBLs, biliary complications, HAT, re-transplantation, blood transfusion, and graft and patient survival

DCD, donation after cardiac death; ITBLs, ischemic-type biliary lesion; HAT, hepatic artery thrombosis.

**Table 2:** Pretransplant characteristics of included studies.

Study	Sample Size (tPA vs. Non-tPA)	Donor Age (Years) (tPA vs. Non-tPA)	MELD Score (tPA vs. Non-tPA)	WIT Functional (Min) (tPA vs. Non-tPA)	CIT (Min) (tPA vs. Non-tPA)
**Seal (2014) [23]**	85 vs. 33	36.3 ± 14.8 vs. 38.0 ± 14.9 (*P* = 0.99)	20.1 ± 8.2 vs. 16.5 ± 10.8 (*P* = 0.38)	21.1 ± 8.3 vs. 23.5 ± 7.6 (*P* = 0.16)	306.0 ± 72.0 vs. 258.0 ± 60.0 (*P* = 0.004)
**Eghtesad (Randomized) (2015) [33]**	11 vs. 12	61.8 ± 5.9 vs. 56.0 ± 11.0 (*P* = 0.13)	22.0 ± 5.0 vs. 23.0 ± 5.0 (*P* = 0.63)	21.0 ± 7.0 vs. 23.0 ± 4.0 (*P* = 0.40)	389.0 ± 36.0 vs. 373.0 ± 76.0 (*P* = 0.53)
**Eghtesad (Non-randomized) (2015) [33]**	35 vs. 22	56.0 ± 9.0 vs. 56.0 ± 11.0 (*P* = 0.99)	22.0 ± 7.0 vs. 23.0 ± 6.0 (*P* = 0.58)	25.0 ± 7.0 vs. 24.0 ± 7.0 (*P* = 0.63)	387.0 ± 68.0 vs. 389.0 ± 107.0 (*P* = 0.93)
**Kubal (2016) [21]**	30 vs. 61	31.5 ± 13.3 vs. 36.2 ± 14.7 (*P* = 0.14)	23.2 ± 8.9 vs. 16.0 ± 5.8 (*P* < 0.001)	19.0 ± 5.2 vs. 26.2 ± 7.2 (*P* = 0.01)	288.0 ± 41.5 vs. 429.0 ± 138.5 (*P* < 0.001)
**Bohorquez (2017) [24]**	100 vs. 38	37.8 ± 14.6 vs. 37.6 ± 14.6 (*P* = 0.95)	20.7 ± 5.4 vs. 20.8 ± 5.7 (*P* = 0.92)	20.4 ± 7.5 vs. 18.7 ± 10.6 (*P* = 0.3)	304.0 ± 92.2 vs. 240.6 ± 45.7 (*P* < 0.001)

Non-tPA, non-tissue plasminogen activator group (non-thrombolytic group); tPA, tissue plasminogen activator group (thrombolytic group); MELD, Model for End-Stage Liver Disease; WIT, Warm ischemia time; CIT, Cold ischemia time.

**Table 3:** Post-transplant outcomes of included studies.

Study	ITBLs (tPA vs. Non tPA)	Total Biliary Complications (tPA vs. Non-tPA)	Bile Leak (tPA vs. Non-tPA)	Anastomotic Strictures (tPA vs. Non-tPA)	HAT (tPA vs. Non-tPA)	Blood Transfusion (pRBC) (tPA vs. Non-tPA)	Graft Survival (1-Year) (tPA vs. Non-tPA)	Patient Survival (1-Year) (tPA vs. Non-tPA)
**Seal (2014) [23]**	2/85 (2.35%) vs. 7/33 (21.21%) (*P* = 0.002)	14/85 (16.47%) vs. 11/33 (33.33%) (*P* = 0.02)	NA	NA	NA	3.2 ± 3.4 vs. 3.1 ± 2.3 (*P* = 0.74)	82/85 (96.47%) vs. 23/33 (69.69%) (*P* < 0.001)	83/85 (97.64%) vs. 29/33 (87.87%) (*P* = 0.08)
**Eghtesad (Randomized + non-randomized) (2015) [33]**	1/34 (2.94%) vs. 1/46 (2.17%) (*P* = 0.83)	NA	NA	NA	1/34 (2.94%) vs. 3/46 (6.52%) (*P* = 0.40)	NA	NA	NA
**Kubal (2016) [21]**	0/30 (0%) vs. 11/61 (18.03%) (*P* = 0.01)	8/30 (26.66%) vs. 43/61 (70.50%) (*P* < 0.001)	1/30 (3.33%) vs. 4/61 (6.51%) (*P* = 0.85)	7/30 (23.33%) vs. 28/61 (45.90%) (*P* = 0.06)	0/30 (0%) vs. 2/61 (3.27%) (*P* = 0.80)	4.2 ± 3.2 vs. 7.2 ± 6.0 (*P* = 0.01)	26/30 (86.67%) vs. 49/61 (80.32%) (*P* = 0.14)	26/30 (86.67%) vs. 53/61 (86.88%) (*P* = 0.90)
**Bohorquez (2017) [24]**	3/100 (3.0%) vs. 2/38 (5.26%) (*P* = 0.63)	25/100 (25.0%) vs. 9/38 (23.68%) (*P* = 0.87)	5/100 (5.0%) vs. 2/38 (5.26%) (*P* = 0.89)	19/100 (19.0%) vs. 5/38 (13.15%) (*P* = 0.42)	3/100 (3.0%) vs. 3/38 (7.89%) (*P* = 0.20)	3.4 ± 4.4 vs. 4.5 ± 3.8 (*P* = 0.16)	92/100 (92.0%) 29/38 (76.31%) (*P* = 0.02)	93/100 (93.0%) vs. 33/38 (86.84%) (*P* = 0.41)

Non-tPA, non-tissue plasminogen activator group (non-thrombolytic group); tPA, tissue plasminogen activator group (thrombolytic group); ITBLs, ischemic-type biliary lesion; HAT, hepatic artery thrombosis; pRBC, packed red blood cells; NA, not applicable.

## Abbreviations

Abbreviations	Full Name
CIT	Cold Ischemia Time
DBD	Donation after Brain Death
DCD	Donation after Circulatory Death
ECD	Extended Criteria Donor
EGD	Early graft dysfunction
HA	Hepatic Artery
HAT	Hepatic Artery Thrombosis
IC	Ischemic Cholangiopathy
I/R	Ischemia-reperfusion
PNF	Primary Non-Function
PV	Portal Vein
MELD	Model for End-Stage Disease
PBG	Peribiliary gland
PVP	Peribiliary Vascular Plexus
PRS	Post reperfusion syndrome
RCT	Randomized Control Trial
SCS	Static Cold Storage
tPA	Tissue Plasminogen Activator
WIT	Warm Ischemia Time
